# Culture optimization of *Streptomyces* sp. KRA16-334 for increased yield of new herbicide 334-W4

**DOI:** 10.1371/journal.pone.0301104

**Published:** 2024-04-09

**Authors:** Young Sook Kim, Kyoung Soo Jang, Jung Sup Choi

**Affiliations:** Eco-friendly and New Materials Research Center, Korea Research Institute of Chemical Technology, Daejeon, South Korea; Benemérita Universidad Autónoma de Puebla: Benemerita Universidad Autonoma de Puebla, MEXICO

## Abstract

This study aimed to isolate actinomycetes that exhibit strong herbicidal activity, identify compounds active against weeds, and researching methods to improve the production of these compounds through culture optimization to establish a foundation for the development of environmentally friendly bioherbicides. 334-W4, one of the herbicidal active substances isolated from the culture broth of *Streptomyces* sp. KRA16-334, exhibited herbicidal activity against various weeds. The molecular formula of 334-W4 was determined to be C_16_H_26_N_2_O_6,_ based on ESI-MS (m/z) and ^1^H and ^13^C NMR spectral data. It had molecular weight 365.1689 [M+Na] and 343.1869 [M+H], indicating the presence of the epoxy-*β*-aminoketone moiety based on HMBC correlations. Additionally, selective culture was possible depending on the addition of trifluoroacetic acid (TFA) during culture with GSS medium. Experiments confirmed that exposure of the KRA16-334 strain to UV irradiation (254 nm, height 17 cm) for 45 seconds improved the yield of the active substance (334-W4) by over 200%. As a result of examining yields of active materials of four mutants selected through optimization of culture conditions such as temperature, agitation, and initial pH, the yield of one mutant 0723–8 was 264.7 ± 12.82 mg/L, which was 2.8-fold higher than that of wild-type KRA16-334 at 92.8 ± 5.48 mg/L.

## Introduction

Among various methods of weed control aiming at improving crop productivity, synthetic herbicides are widely used worldwide primarily due to their relatively low production costs and ability to provide effective control. However, various problems are emerging due to continuous use of organic synthetic herbicides. There problems include carcinogenic classification of glyphosate, a non-selective herbicide, safety issues such as human toxicity problems of paraquat, and emergence of resistant weeds due to long-term and continuous use of the same herbicide. Furthermore, due to commercialization of GMO (genetically modified organism) crops with glyphosate resistance, the use of herbicides other than glyphosate is decreasing. Even newly developed crop protection products are facing stricter global registration regulations in recent years. Therefore, the development of new herbicides with environmental friendliness and creative structures with new mode of action is needed to solve problems related to synthetic herbicides. However, since the development of 4-hydroxyphenylpyruvate dioxygenase (HPPD) inhibitors [1–3] such as benzobicyclon, mesotrione, and tefuryltrione, no herbicides with a new mode of action have been developed yet. Thus, it is necessary to move away from the existing imitation technology and develop a new concept herbicide. Unfortunately, no clear development results have been reported so far. In this situation, various attempts are being made worldwide to develop new herbicides, with a growing interest in natural products. Natural product-based herbicides are currently being actively explored through direct use of various natural substances or investigation of new substances with optimal chemical structures to act as lead compounds for developing novel synthetic compounds with unique functional mechanisms [4].

Actinomycetes produce a wide variety of physiologically active substances. Some of them are known to exhibit strong bioherbicidal activities. Herbicidal active substances discovered in actinomycetes so far include bialaphos produced by *Streptomyces viridochromogenes* or *S*. *hygroscopicus* [5, 6] and hydantocidin produced by *S*. *hygrioscopocus* [7]. In addition, there are thaxtomin A produced by *S*. *scabies* [8], actinonin produced by Actinomyces MG848-hf6, and albucidin, an active substance with herbicidal activity that causes bleaching from *S*. *albus* [9]. Secondary metabolites produced by actinomycetes mainly consist of substances such as amino acids, peptides, nucleosides, macrolides, lactones, amides, and amines [10]. Among these, compounds with potent herbicidal activities, such as Bialaphos [11, 12] and Herbicidins [13, 14], have been developed as commercial herbicides or are currently being studied. In addition, diverse structures and potent herbicidal activities of actinomycete secondary metabolites may offer not only direct applications as bioherbicides, but also potential for the development of synthetic herbicides with novel target sites. In particular, *Streptomycetes* sp. have the potential to produce new physiologically active substances. By exploring candidate materials as herbicides, new substances with different mechanisms of action compared to existing herbicides might be developed [15]. Although active metabolites derived from actinomycetes show strong herbicidal activities, their productivity is low, which limits their industrial use. The productivity of actinomycete secondary metabolites is closely related to the optimization of culture conditions, including strain improvement. The technology for high-yield strain development and fermentation processes is recognized as the most important factor in the development of bioherbicides.

Therefore, this study was conducted to establish a foundation for the development of environmentally friendly bioherbicides. This was accomplished by isolating actinomycetes that showed strong herbicidal activity, identifying active compounds, and researching methods to improve the productivity of these compounds through culture optimization.

## Materials and methods

### Isolation and culture of soil actinomycetes

*Streptomyces* sp. KRA16-334 isolate was separated and cultured using the method described previously [16]. M3 medium (1% soytone, 1% glucose, 2% soluble starch, 0.3% CaCO_3_, 0.02% FeSO_4_, 0.1% antifoaming agent, 1 L water) was used for mass culture to isolate active substances and for selecting culture mutants. The culture was incubated on a rotary shaker (160 rpm) for 7 days at 27°C. It was then centrifuged at 8,000 rpm for 10 minutes at 4°C. After cells were removed, the culture filtrate was used to evaluate herbicidal activity.

### *In vivo* assay for herbicidal activity

For the evaluation of herbicidal activity, seeds of five grass weeds (*Sorghum bicolor*, *Echinochlia crus-galli*, *Agropyron smithii*, *Digitaria ciliaris*, *Panicum dichotomiflorum*) and five broad-leaf weeds (*Solanum nigrum*, *Aeschynomene indica*, *Abutilon avicennae*, *Xanthium strumarium*, *Calystegia japonica*) were grown under greenhouse conditions using the methods described previously [17]. The culture filtrate and active compounds of each concentration (14 mL per pot) were applied to the seedlings using a laboratory spray gun 12 days after sowing seeds. The herbicidal activity through foliar application was evaluated at 7 days after treatment by visually determining the percentage of injury (0%, no damage; 100%, complete control). All experiments were independently repeated three times. The mean percentage of injury was calculated based on three replicates. Distilled water was used as an untreated control.

### Identification of strain KRA16-334

Taxonomic identification of KRA16-334 bacteria was performed by 16S rRNA sequencing. Multiple sequence alignment and molecular phylogenetic analysis were carried out using BioEdit and MEGA-X software programs. For PCR amplification, primers 27F (5′-AGGRTGARCCTGCTCAG-3′) and 1492R (5′-GGRTACCTTGRACGACTT-3′) were used. Sequences of obtained 16S rRNA genes were analyzed using BLASTN program on the NCBI website (http://www.ncbi.nlm.nih.gov).

### Purification and structural identification of active compounds

Culture filtrates were passed through a Diaion HP-20 column (15 cm × 50 cm) (Adsorbent resin from Mitsubish Co., Ltd, and washed with H_2_O (5.0 L) followed by MeOH (5.0 L). After 50% MeOH fraction was evaporated to dryness under vacuum, the crude extract (23.4 g) was subjected to reversed phase C-18 (from Merck) flash chromatography (5 cm × 50 cm) using a solvent system of H_2_O and MeOH with a 10% increase of MeOH to yield six fractions (MeOH/H2O = 0/100 ∼ 50/50). The 10% MeOH fraction was purified by Sephadex LH-20 column chromatography using 50% aq. MeOH as the eluting solvent and separated by preparative HPLC (high-performance liquid chromatography) on an ODS C_18_ reverse-phased silica gel column (Atlantis T3, 5μm, 10 x 250mm; WATERS) using 30% MeOH over 60 minutes at a flow rate of 6 ml minute^-1^. Additionally, the 70% MeOH fraction (1.75 g) of HP20 resin was isolated by C_18_ sep-pak catridge (MeOH/H2O = 20/80∼100/0) and purified by HPLC using 55% acetonitrile containing 0.02% trifluoacetic acid. For herbicide activity evaluation, each fraction was concentrated using a pressure reducing concentrator (EYELAS SB-1200, EYELA, Shanghai, China) and then diluted to the undiluted concentration and examined for herbicide activity using *D*. *ciliaris*.

The chemical structure of the active material was identified via molecular weight measurement using ESI–MS (SYNAPT G2, Waters) and NMR analysis of ^1^H, ^13^C NMR, 1H–1H COSY, HMBC, HSQC, NOESY, and ROESY (Bruker AVANCE HD 700 NMR spectrometer, 700 MHz for ^1^H and 176 MHz for ^13^C). NMR spectra were measured by dissolving in DMSO-d_6_, CD_3_OD. Chemical shifts were referenced to the residual solvent signal (δ_C_ 39.5 and δ_H_ 2.50, δ_C_ 49.15 and δ_H_ 4.78).

### Changes in yields of active compounds based on culture method

KRA16-334 was inoculated into M3 and GSS media (glucose 20, soluble starch 10, soybean meal 25, beef ext. 1, yeast ext. 4, NaCl 2, KH_2_PO_4_ 0.25, CaCO_3_ 2 g/L) in 100 ml/500 ml baffled flasks and cultured in a rotary shaker (160 rpm) at 27°C. After 3 days of incubation, 200 μL of trifluoroacetic acid (TFA) was added to each culture at 1-day intervals. The culture was then incubated for an additional 10 days. The yield of the active substance was determined and verified by conducting HPLC (Atlantis T3 column, 5 μm, 10x250 mm; WATERS) analysis of the culture filtrate using 0∼100% acetonitrile containing 0.02% TFA over a period of 45 minutes at a flow rate of 0.8 mL/minute. Additionally, the herbicidal activity against *D*. *ciliaris* was evaluated.

### UV mutagenesis for active compound 334-W4 yield-up

Mutagenesis for yield-up of an active strain was carried out according to the method described previously [18]. KRA16-334 spore suspensions (1.2 x 10^3^) were smeared onot Bennett agar plates, followed by exposure to UV light (254 nm, 17 cm distance) under dark conditions for 30 seconds to 45 seconds. Bacterial cells mutagenized by UV exposure were incubated at 27°C for 5 days until colonies became visible. Survival and lethality rates were then assessed. Survival rate was calculated by dividing the number of colonies of UV-exposed plates by the number of colonies of plates without UV treatment. A UV exposure time that resulted in a 1% survival rate was determined through repeated experiments and subsequently utilized for mutagenesis to select high-yield mutants (S1 Table in S1 File).

Surviving mutant colonies were cultured in M3 liquid medium for 7 days following the previously described method (27°C, 160rpm). Subsequently, cultures were centrifuged (8,000rpm, 10min) and the supernatant was utilized for activity and yield evaluation. The herbicidal activity of each mutant was evaluated using *D*. *sanguinalis* as previously described. Quantitative analysis was conducted to investigate the content of active compound 334-W4 in the culture under the following HPLC conditions: column: WATERS Atlantis T3 column (i.d. 4.6 x 250 mm); mobile phase: aq. 30% aqueous MeOH; flow rate: 0.8 mL/min; detection wavelength, 210 nm; and retention time: 30 minutes. After confirming the reproducibility of high-yield mutants first selected through subculture, mutants showing stable production were finally selected.

### Culture optimization of selected mutants

Selected mutants used M3 liquid medium as basal medium. To establish optimal culture conditions, different culture temperatures, agitation speeds, and initial medium pH values were used. Content of active compound in culture broth was investigated by HPLC quantitative analysis. Mutants were incubated under the following conditions: 1) incubation temperature at 15, 20, 27, 30 or 35°C; 2) agitation speed of 50 to 250 rpm; and 3) initial medium pH of 3, 5.5, 7, 8.5, or 10 adjusted using 1 N HCl and 1 N NaOH. Mutants were then cultured for 7 days. Culturing under each condition was replicated three times.

### Statistical analyses

All treatments were statistically analyzed using one-way ANOVA (Analysis of Variance) in OriginPro 8.1 (OriginLab, 2021). Fisher’s LSD test (*p* < 0.05) was used to compare means.

## Results

### Classification and herbicidal activity of strain KRA16-334

**Fig 1:**
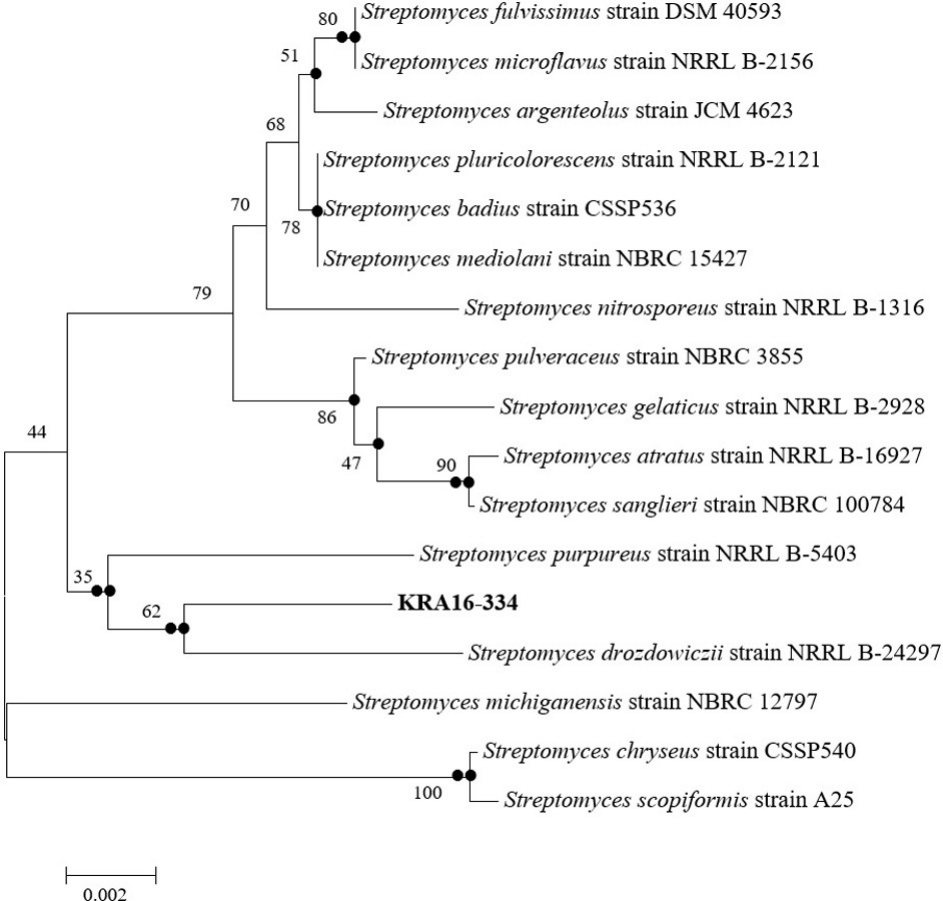
Neighbor-joining tree of strain KRA16-334 based on its 16S rRNA gene sequence, showing phylogenetic relationship with related *Streptomyces* species. Numbers at each branch node indicate bootstrap percentage of 1000 replications (S1 Fig in S1 File).

**Fig 2:**
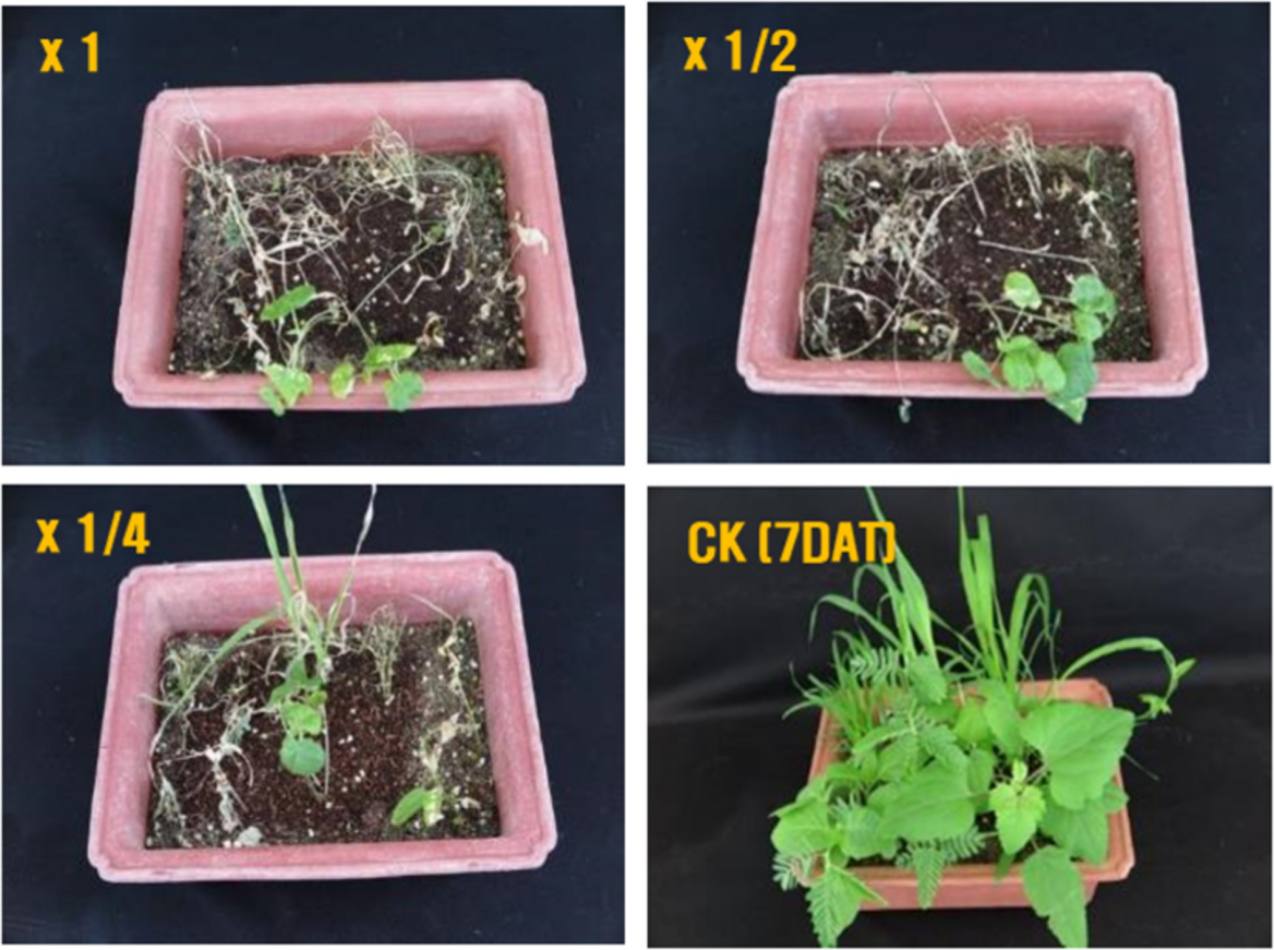
Herbicidal activities of *Streptomyces* sp. KRA16-334 culture filtrate against five broadleaf and five grass weed species under greenhouse conditions at 7 days after treatment (DAT).

The 16S rRNA sequence of strain KRA16-334 shared 99% similarities with 16S rRNA sequence of *Streptomyces drozdowiczii* strain NRRL B-24297 (Fig 1). Thus, it was named *Streptomyces* sp. KRA16-334. After culturing the KRA16-334 strain in M3 medium and evaluating herbicidal activities against 10 weeds using the culture filtrate, it was observed that the 2-fold dilution applied to leaves resulted in complete control of all weed species except for *A*. *avicenae*. In addition, when the culture filtrate was diluted four times and applied, it resulted in complete control (100%) for seven types of weeds except for *S*. *bicolor*, *A*. *avicenae*, and *C*. *japonica*. It also demonstrated more than 80% herbicidal activities against three weeds (Fig 2). Phytotoxic symptoms caused by KRA16-334 appeared within 24 h after culture broth foliage treatment, indicating that KRA16-334 acted very quickly. No weeds regenerated even at 7 days after treatment, suggesting a residual activity of KRA16-334. Major phytotoxic symptoms were wilting or leaf burn-down and stunting with eventual plant death.

### Chemical properties and structure determination

Herbicide compounds 334-100M and 334-W4 were separated from the culture filtrate of *Streptomyces* sp. KRA16-334. 334-100M was isolated from the 100% MeOH fraction when adsorbed to HP20 resin. It was purified by C_18_ sep-pak cartridge and preparative high-performance liquid chromatography (prep HPLC). As a result of NMR analysis, 334-100M was identified as herboxidien, which contained a tetrahydropyran acetic acid moiety and a side chain with a conjugated diene. It had a molecular weight of 438.6 and a molecular formula of C_25_H_42_O_6_. Wideman et al. (1992) have reported that heboxidiene is a novel polyketide that exhibits potent herbicidal properties against several annual weed species [19]. In addition, a study on the enhancement of herboxidiene productivity through gene regulation of *Streptomyces chromofuscus* ATCC 49982 has been reported [20].

**Fig 3:**
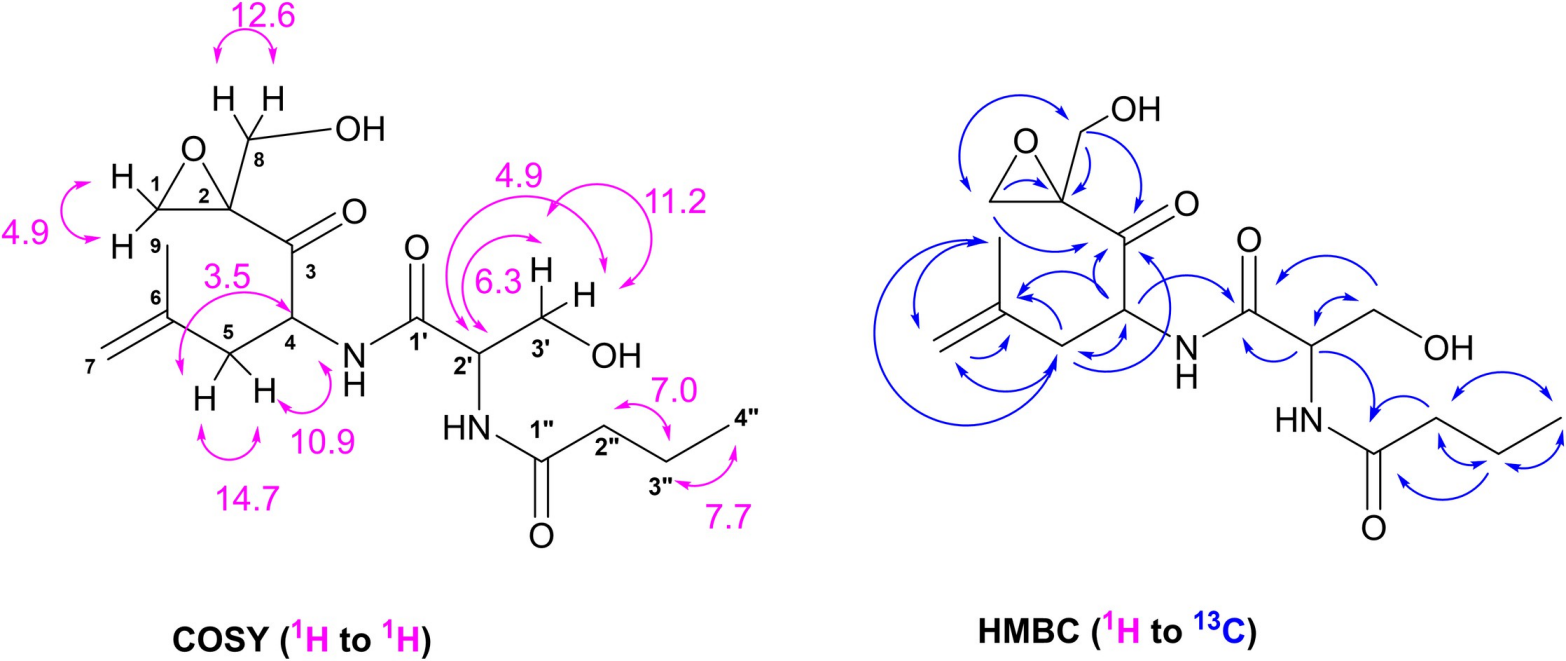
Structure of herbicidal compound 334-W4 produced by KRA16-334 (in DMSO-d_6_, CD_3_OD) (S2−S8 Figs in S1 File).

334-W4 was isolated from the 50% MeOH fraction of HP20 CC. It is a novel herbicidal compound. It exhibited strong activities against broad-leaved weeds. Regarding chemical properties of 334-W4, it is a colorless oil that is soluble in H_2_O, DMSO, MeOH, and EtOH. It had an end absorption UV λmax (MeOH) and ESI-MS (m/z) Calcd 365.1689 (M+Na), 343.1869 (M+H). Its molecular formula was C_16_H_26_N_2_O_6_. The structure of 334-W4 was determined by analyzing ESI-MS, ^l^ H and ^13^C NMR, and 2D NMR spectral data. Its ^13^C NMR spectrum displayed 16 signals, including two -CH_3_, six -CH_2_-, one = CH_2_, two >CH-, two quaternary carbons, and three carbonyl carbons (Table 1). ^1^H-^1^H COSY NMR analysis confirmed four sequential protons: -^8^CH_2_-OH, -NH-^4^CH-^5^CH_2_-, -NH-^2’^CH-^3’^CH_2_-OH, and -^2"^CH_2_-^3"^CH_2_-^4"^CH_3_ (Fig 3). The presence of the epoxy ring was indicated by chemical shift of C-1 methylene (δ_C_ 48.0, δ_H_ 3.07). The connection of these structural fragments was deduced from observation of HMBC correlations as shown in Fig 3. Correlations from C-3 (δ 206.5) to H-4, H-5, H-l, and H-8; and from C-2 (δ 63.0) to H-l and H-8 (Fig 3), along with the presence of the epoxy ring indicated the presence of the epoxy-*β*-aminoketone moiety. As a result of determining the structure of 334-W4 by 1D and 2D NMR and MS spectral analysis, it was identified as TMC-86A reported previously [17]. TMC-86A isolated from fermentation broth of *Streptomyces* sp. TC 1084 has been reported as a novel 20S proteasome inhibitor with an epoxy-*β*-aminoketone moiety [21]. However, its biological activity such as herbicidal activity has not been reported yet.

**Table 1:** ^1^H and ^13^C NMR data of 334-W4 (in DMSO-d_6_).

Position	δ_C_	δ_H_
1	48.0	3.07 (s)
2	63.0	
3	206.5	
4	50.2	4.54 (dd, 10.8, 3.5)
5	36.6	2.07 (dd, 9.8, 2.8), 2.41 (dd, 14.0, 2.8)
6	141.0	
7	113.2	4.74 (br s), 4.78 (br s)
8	59.5	3.37 (d, 12.6), 4.08 (d, 12.6)
9	22.0	1.70 (s)
1’	170.3	
2’	54.6	4.31 (t, 6.3)
3’	61.6	3.45 (m), 3.55 (m)
1"	172.1	
2"	37.0	2.10 (t, 7.0)
3"	18.6	1.49 (sextet, 7.0)
4"	13.6	0.80 (t, 7.7)
4-NH		7.97 (d, 7.7)
8-OH		5.07 (t, 6.3)
2’-NH		7.76 (d, 7.7)
3’-OH		4.78 (overlapped)

The herbicidal compound 334-W4 (TMC-86A) showed higher activities against broad-leaf weeds than grass weeds. At a concentration of 1000 mg/L, 344-W4 showed 100% control effect on broadleaf weeds except for *A*. *avicenae* and on grass weeds *S*. *bicolor*, *D*. *sanguinalis*, *P*. *dichotomifloru*. It showed herbicidal activities of 83.3% and 66.7% against *E*. *crus-galli* and *A*. *smithii*, respectively (Table 2).

**Table 2:** Herbicidal activities of 334-W4 (TMC86-A) against major weeds through foliar application in greenhouse conditions.

Weeds		Herbicidal activity (%)^x^			
		2000	1000	500	250 (mg/L)
Grass	SORBI	100^**y**^ ± 0 BC^**z**^	100 ± 0 BC	46.7 ± 9.5 B	10 ± 5.0
	ECHCG	100 ± 0	83.3 ± 5.7 BC	35 ± 7.6 B	0 ± 0
	AGRSM	100 ± 0	66.7 ± 8.6 B	30 ± 11.5 B	10 ± 12.5 B
	DIGSA	100 ± 0 BC	100 ± 0 BC	88.3 ± 10.4 BC	45 ± 8.6 B
	PANDI	100 ± BC	100 ± 0 BC	60 ± 13.3 B	30 ± 11.5 B
Broad-leaf	SOLNI	100 ± 0 BC	100 ± 0 BC	100 ± 0 BC	60 ± 5.9 BC
	AESIN	100 ± 0 BC	100 ± 0 BC	100 ± 0 BC	75 ± 13.2 BC
	ABUTH	67.4 ± 5.7 BC	45 ± 7.6 B	20 ± 5.0 B	5.7 ± 10.4
	XANSI	100 ± 0 BC	100 ± 0 BC	100 ± 0 BC	95 ± 0.6 BC
	CAGHE	100 ± 0 BC	100 ± 0 BC	100 ± 0 BC	90 ± 3.5 BC

Weeds name (Bayer code): SORBI, *Sorghum bicolor*; ECHCG, *Echinochlia crus-galli*; AGRSM, *Agropyron smithii*; DIGSA, *Digitaria sanguinalis*; PANDI, *Panicum dichotomiflorum*; SOLNI, *Solanum nigrum*; AESIN, *Aeschynomene indica*; ABUTH, *Abutilon avicennae*; XANSI, *Xanthium strumarium*; CAGHE, *Calystegia japonica*.

^x^Herbicidal activity was determined at 7 days after treatment.

^y^Herbicidal activity (%) was determined as visual injury percentage (0%: no injury, 100%: complete death). Values are means with SD of triplicate experiments. All experiments were independently repeated three times.

^z^B: stunting; C: desiccation or leaf burn-down.

### Changes in yields of active compounds based on culture method

**Fig 4:**
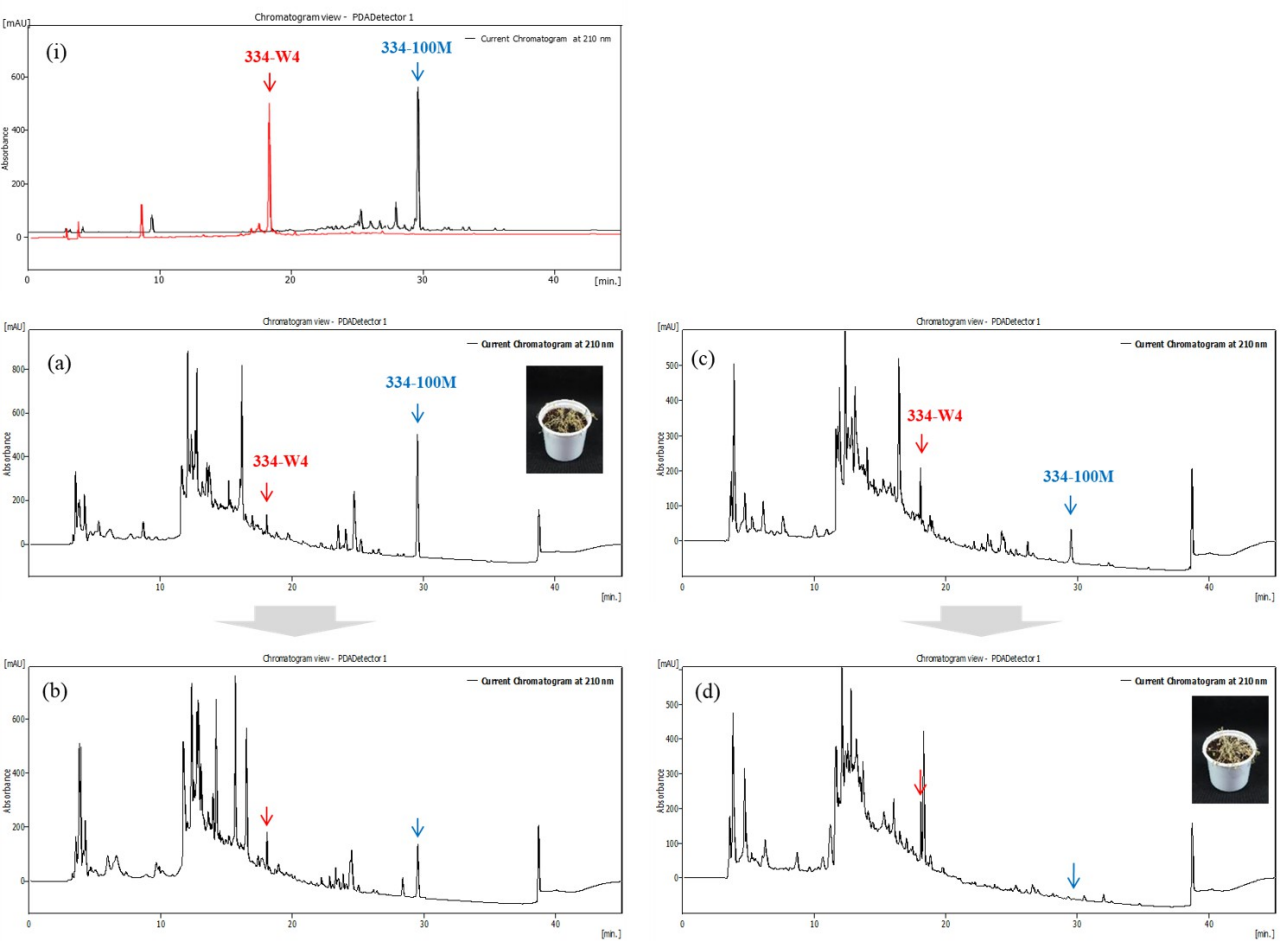
Changes in yields of active compounds based on culture method. (a) M3 medium, (b) M3+TFA, (c) GSS medium, (d) GSS+TFA. TFA was added on the 5th day of incubation and the culture medium was analyzed by HPLC after a total of 8 days of incubation. Column, Atlantis T3 column (5 μm, 10 x 250 mm; WATERS); mobile phase, 0∼100% aq. acetonitrile/0.02% TFA; flow rate, 0.8 mL/min; detection wavelength, 210 nm.

To decrease the production of compound 334-100M reported to have herbicidal activity and to selectively cultivate the new active substance 334-W, KRA16-334 was cultured in the selective medium GSS. Comparison of culture broth using M3 and GSS media revealed that M3 medium resulted in significantly higher productivity of 334-100M than GSS medium. With the GSS medium, the yield of 334-W4 was increased, while that of 334-100M showed a relative decrease (Fig 4A and 4C). Additionally, it was confirmed that the productivity of the active material 334-100M was significantly reduced during culture when TFA was added to the medium throughout the culturing process. Particularly, adding TFA to the GSS medium on the 5th day resulted in no production of 334-100M, whereas the yield of 334-W4 was increased significantly (Fig 4).

### Effects of culture conditions of selected mutants on yield of 334-W4 (TMC-86A)

**Fig 5:**
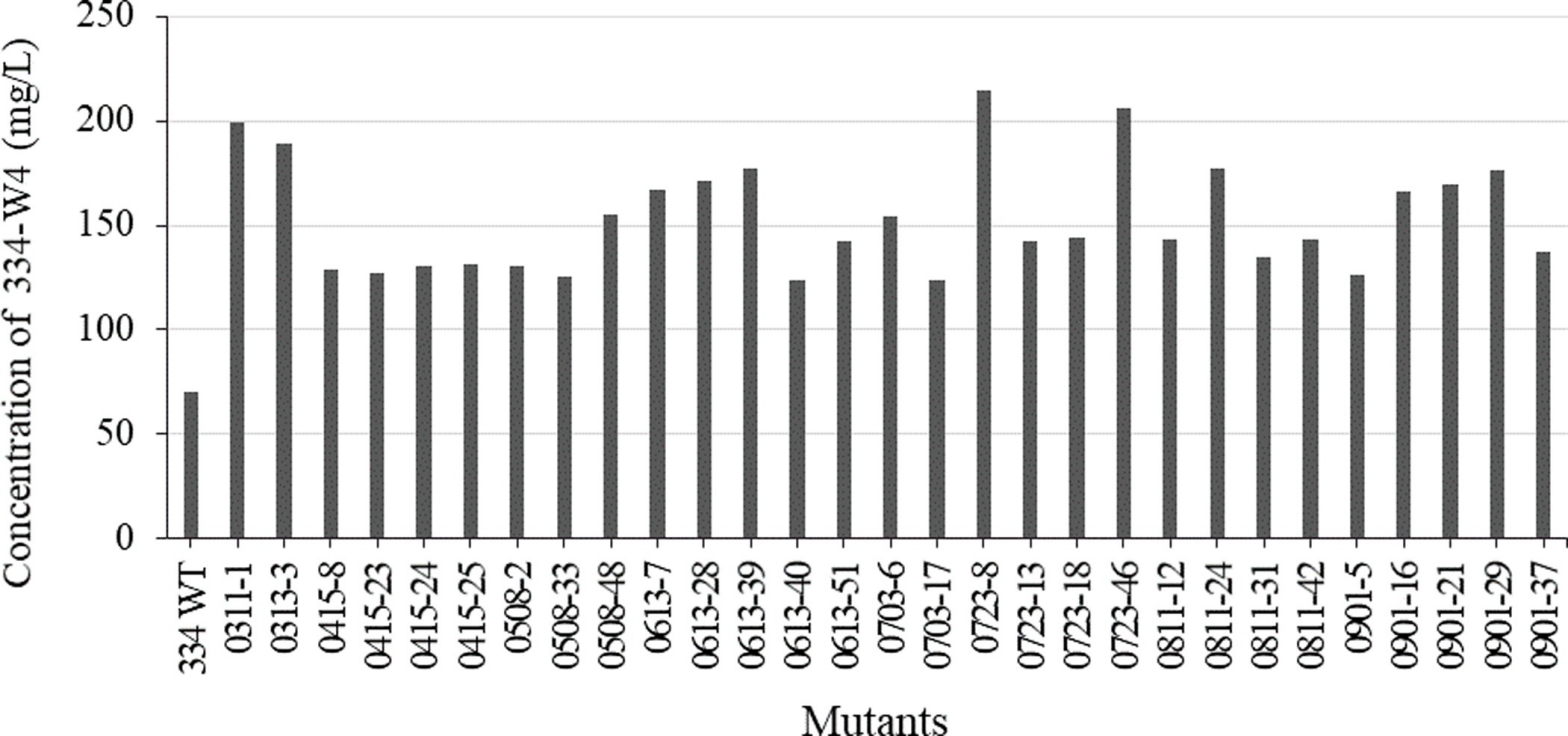
Selection of UV mutants to increase the yield of herbidcidal compound 334-W4.

**Fig 6:**
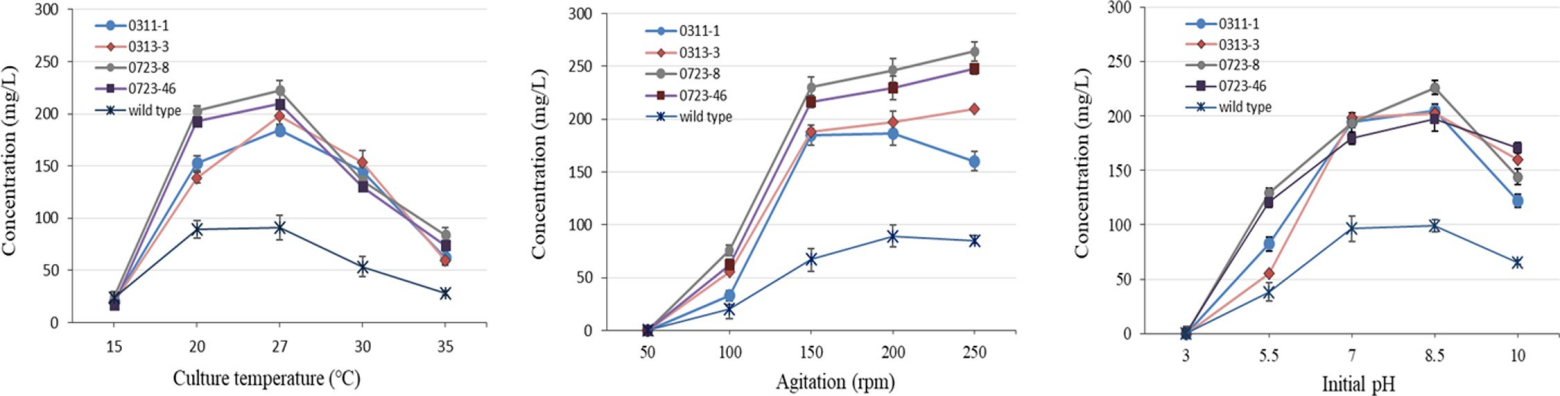
Culture optimization of selected mutant strains to increase the productivity of herbicidal compound 334-W4. Vertical bar indicates standard error of the mean. All experiments were independently repeated three times (S2−S4 Tables in S1 File).

Strain improvement strategies, especially mutagenesis and screening of hyper-producing mutants, are very important in the production of secondary metabolites during the fermentation process [22]. In this study, *Streptomyces* sp. KRA16-334 was exposed to UV for different time periods. To optimize the quantity of mutagens, survival rate was calculated. When UV treatment was performed for 45 sec, its survival rate was 1.2 ± 0.57%. Screening of various mutants was performed based on 334-W4 (TMC-84A) yield. The wild type produced 69.9 mg/L of 334-W4, while 45 sec UV-treated mutants produced up to 214.7 mg/L of 334-W4 (Fig 5). Among UV mutants tested, four mutants with high yields of 334-W4 were selected. The productivity of 334-W4 by culture temperature, agitation speed, and initial pH was then investigated. As a result of examining changes in yield according to culture temperature, the productivity of 334-W4 was excellent at 20°C to 27°C. It tended to decrease rapidly at 30°C (Fig 6). In addition, the yield of the selected mutant was higher than that of the wild type at a stirring speed of 150 rpm or more and an initial pH of 7 to 8.5 (Fig 6). *Streptomyces* sp. KRA16-334 and its mutants were able to grow even under alkaline conditions of pH 10. As the agitation speed increased, the productivity of the active compound 334-W4 tended to increase. Among the four selected mutants, the yield of mutant 0723–8 (264.7 ± 12.82 mg/L) was 2.8 times higher than that of the wild type KRA16-334 (92.8 ± 5.48 mg/L). It was the highest at a temperature of 27°C, an agitation speed of 250 rpm, and an initial pH of 8.5.

## Discussion

Secondary metabolites produced by *Streptomyces* sp. are being studied as potential bioherbicides or herbicide adjuvants in order to develop environmentally friendly agents that can be easily degraded with a low toxicity. In addition, isolation and structural identification of native herbicide-active compounds from *Streptomyces* sp. have been proven to be an effective method for discovering new leads in herbicide development. Therefore, numerous isolation and screening attempts have been made to discover microbial metabolites with bioherbicidal potential for *Streptomycetes*. In this study, we selected KRA16-334, which exhibited herbicidal activities against several weeds, and identified it using 16S rRNA gene sequencing analysis. Among molecular characterization techniques, 16S rRNA gene sequence analysis is a more suitable and time-saving approach to identify *Streptomyces* [23]. The 16S rRNA gene sequence of the selected KRA16-334 strain shared 99% sequence similarities with the16S rRNA gene of *S*. *drozdowiczii* strain NRRL B-24297. Thus, it was named *Streptomyces* sp. KRA16-334. Characteristics of herbicidal activity, include wilting, leaf burn-down, stunting, and ultimately plant death, were then observed after applying KRA16-334 culture filtrate to leaves.

334-100M and 334-W4 were herbicidal active substances isolated from the fermentation broth of *Streptomyces* sp. KRA16-334. 334-100M was heboxidiene with a molecular weight of 438.6 and a molecular formula of C25H42O6. Wideman et al. (1992) have reported that heboxidiene is a novel polyketide that exhibits potent herbicidal properties against several annual weed species [19]. In addition, Jha et al. (2014) have reported that herboxidiene productivity can be enhanced through gene regulation of *Streptomyces chromofuscus* ATCC 49982 [20]. 334-W4 was identified as TMC-86A having a molecular formula of C16H26N2O6. TMC-86A, a novel 20S proteasome inhibitor with an epoxy-β-aminoketone moiety, has been reported previously [21]. However, its biological activity such as herbicidal activity has not been reported yet.

Addition of TFA to culture medium during culturing had a significant impact on the production of active compounds (Fig 4). The productivity of 334-100M with the M3 medium was significantly higher than that with the GSS medium. With the GSS medium, the yield of 334-W4 was increased whereas that of 334-100M showed a relative decrease. Adding TFA to the GSS medium on the 5th day resulted in no production of 334-100M, although the yield of 334-W4 was increased significantly. These findings suggest that manipulating culture conditions, specifically by using GSS medium and adding TFA, can influence the production of active substances, offering insights for potential herbicidal applications. The ability of *Streptomyces* sp. to produce active substances can be greatly increased or completely lost under various nutritional and culturing conditions. Thus, it is not a fixed characteristic [24].

Productivity improvement of secondary metabolites produced by *Streptomyces* sp. depends on optimization of culture conditions, induction of high-yield mutations, and metabolic engineering. In particular, the selection of mutants and the screening of overproducing mutants through appropriate strain-improvement techniques are critical for the production of secondary metabolites during the fermentation process. To have enough production of the herbicidal compound 334-W4, the most important and convenient physical method for obtaining a wide spectrum of mutations was using UV radiation. To achieve powerful and effective mutation selection through UV radiation, a high lethality rate is required [25]. Therefore, four mutant strains with increased productivity of 334-W4 were selected through 45 seconds of UV irradiation at a height of 17 cm. Regarding the influence of culture temperature, the mutant strain showed excellent productivity of 334-W4 at 20°C and 27°C. However, a significant decrease in productivity was observed at temperatures below 15°C and above 30°C. As a result, mutants exhibited a restricted range of culturing temperatures for the production of 334-W4. It was determined that the optimal culture temperature for achieving maximum production of 334-W4 was 27°C. The optimal initial pH for the production of secondary metabolites by *Streptomyces* sp. was found to be neutral pH, which was reported in several studies [26, 27]. Mutants selected through experiments were found to produce the optimal active substance when the pH level was between 7 and 8.5. It was also observed that the maximum productivity of the active substance was achieved at an agitation speed of 250 rpm. This indicates that the production of the active substance 334-W4 by mutants is related to an increase in agitation speed. Agitation was found to have a direct impact on the mixing of nutrients and the amount of dissolved oxygen in the medium, which is crucial for the production of 334-W4. Through studies aimed at removing toxic substances by modifying cultural methods and increasing the yield of the active substance 334-W4 through UV mutation and cultural optimization, *Streptomyces* sp. KRA16-334 culture medium is believed to offer an opportunity for the development of bioherbicides for direct use. Additionally, herbicide compound 334-W4 is considered a promising candidate for the development of novel herbicides and synthetic herbicides with new mechanisms.

## Conclusions

Structural diversity and potent herbicidal activity of actinomycete secondary metabolites not only can be directly used as herbicides, but also can provide opportunities for the development of synthetic herbicides targeting new sites. In this study, we isolated and cultured *Streptomyces* sp. KRA16-334, which exhibited strong herbicide activities. Yield improvement and selective culturing of the herbicidal compound 334-W4 were achieved through UV mutation, culture optimization, and modification of culture medium. The yield of the active compound 334-W4 from Mutant 0723–8 (264.7 ± 12.82 mg/L) selected through UV mutation increased by 2.8-fold compared to that of the wild type KRA16-334 (92.8 ± 5.48 mg/L). The highest yield was observed at a temperature of 27°C, an agitation speed of 250 rpm, and an initial pH of 8.5. In addition, the strain 334-W4 was able to be selectively cultured by adding TFA during culturing in GSS medium. Therefore, the culture broth of *Streptomyces* sp. KRA16-334 can be utilized as a safe bioherbicide. In addition, the herbicidal compound 334-W4 can provide an opportunity for the development of new herbicides.

## Supporting information

S1 File Structures and spectra of compound 334-W4 and UV mutagenesis for active compound 334-W4 yield-up. (DOCX)
