# Supplementary material for: Culture optimization of Streptomyces sp. KRA16-334 for increased yield of new herbicide 334-W4
Source: PLoS One. 2024 Apr 9;19(4):e0301104. doi: 10.1371/journal.pone.0301104 (PMC11003667; doi:10.1371/journal.pone.0301104)
Supplement: S1 File — (DOCX) [file pone.0301104.s001.docx]

Culture optimization of *Streptomyces* sp. KRA16-334 for increased yield of new herbicide 334-W4

Short title: 334-W4(TMC-86A), a new herbicidal compound

Young Sook Kim^1*^, Kyoung Soo Jang^1^, Jung Sup Choi^1*^

^1^Eco-friendly and New Materials Research Center, Korea Research Institute of Chemical Technology, Daejeon, South Korea

*** Corresponding authors:** Young Sook Kim ([gtryoung@krict.re.kr](mailto:gtryoung@krict.re.kr)), Jung Sup Choi ([jschoi@krict.re.kr](mailto:jschoi@krict.re.kr))

# ABSTRACT

This study aimed to isolate actinomycetes that exhibit strong herbicidal activity, identify compounds active against weeds, and researching methods to improve the production of these compounds through culture optimization to establish a foundation for the development of environmentally friendly bioherbicides. 334-W4, one of the herbicidal active substances isolated from the culture broth of Streptomyces sp. KRA16-334, exhibited herbicidal activity against various weeds. 334-W4 was identified as a molecular formula of C16H26N2O6 with ESI-MS (m/z) calculated values of 365.1689 (M+Na) and 343.1869 (M+H), which contains an epoxy-β-aminoketone moiety, through 1D and 2D NMR and MS spectral analysis. Additionally, selective cultivation was possible depending on the addition of trifluoroacetic acid (TFA) during GSS medium cultivation. It was confirmed through experiments that exposure to UV irradiation (254 nm, height 17 cm) for 45 seconds to the KRA16-334 strain improved the yield of the active substance (334-W4) by 200% over. As a result of examining the yield of active materials of four mutants selected through optimization of culture conditions such as temperature, agitation, and initial pH, the yield of Mutant 0723-8 (264.7 ± 12.82 mg/L) increased 2.8-fold compared to that of wild-type KRA16-334 (92.8 ± 5.48 mg/L).

# KEYWORDS

*Streptomyces drozdowiczii* / Herbicidal activity/ TMC-86A /UV mutation/ Optimization

Supporting information

**Structures and spectra of compound 334-W4 and UV mutagenesis for active compound 334-W4 yield-up**

**Contents**

Figure S1. 16S rRNA sequence of *Streptomyces* sp. KRA16-334.

Figure S2. ^1^H (700 MHz) and ^13^C (176 MHz) assignments in CD_3_OD-*d*_6_ of 334-W4.

Figure S3. ^1^H (700 MHz) and ^13^C (176 MHz) assignments in DMSO-*d*_6_ of 334-W4.

Figure S4. ^1^H-NMR (DMSO-*d*_6,_ 700 MHz) spectrum of 334-W4.

Figure S5. ^13^C-NMR (DMSO-*d*_6,_ 176 MHz) spectrum of 334-W4.

Figure S6. ^1^H-NMR (CD_3_OD_,_ 700 MHz) spectrum of 334-W4.

Figure S7. ^13^C-NMR (CD_3_OD_,_ 176 MHz) spectrum of 334-W4.

Figure S8. (+)ESI-MS spectrum of 334-W4.

**Table S1.** UV mutagenesis for active compound 334-W4 yield-up.

**Table S2.** Yield change of active compound 334-W4 according to culture temperature.

**Table S3.** Yield change of active compound 334-W4 according to agitation (raw data).

**Table S4.** Yield change of active compound 334-W4 according to initial pH (raw data).

**
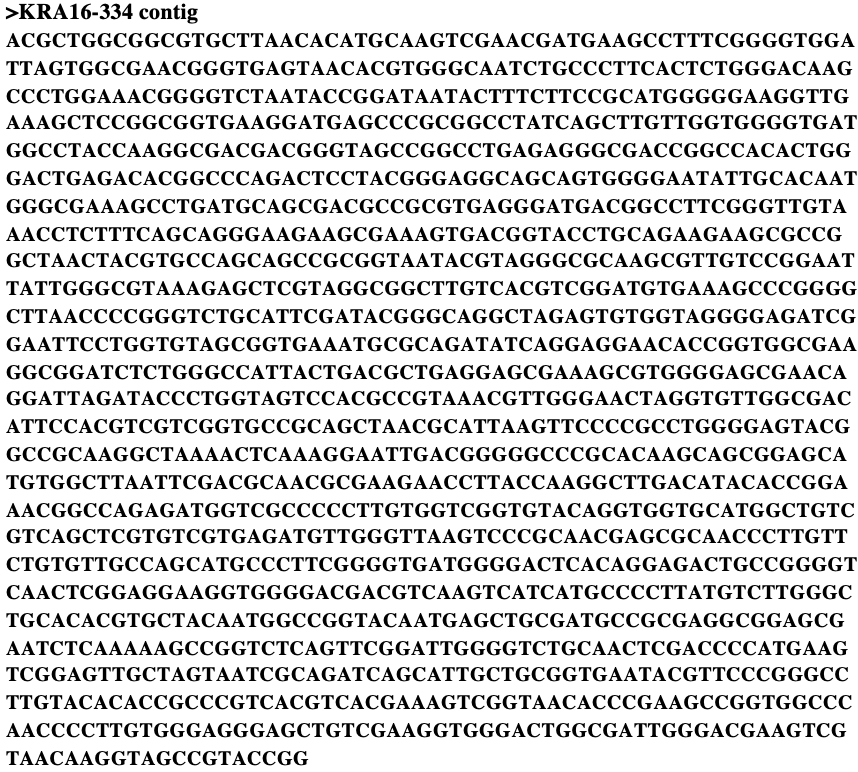
**

Figure S1. 16S rRNA sequence of *Streptomyces* sp. KRA16-334 ([GenBank: PP45541)](https://www.ncbi.nlm.nih.gov/nuccore/PP455413)

Fig. S2. ^1^H (900 MHz) and ^13^C (226 MHz) assignments in CD_3_OD-*d*_6_ of 334-W4.

Fig. S3. ^1^H (900 MHz) and ^13^C (226 MHz) assignments in DMSO-*d*_6_ of 334-W4.


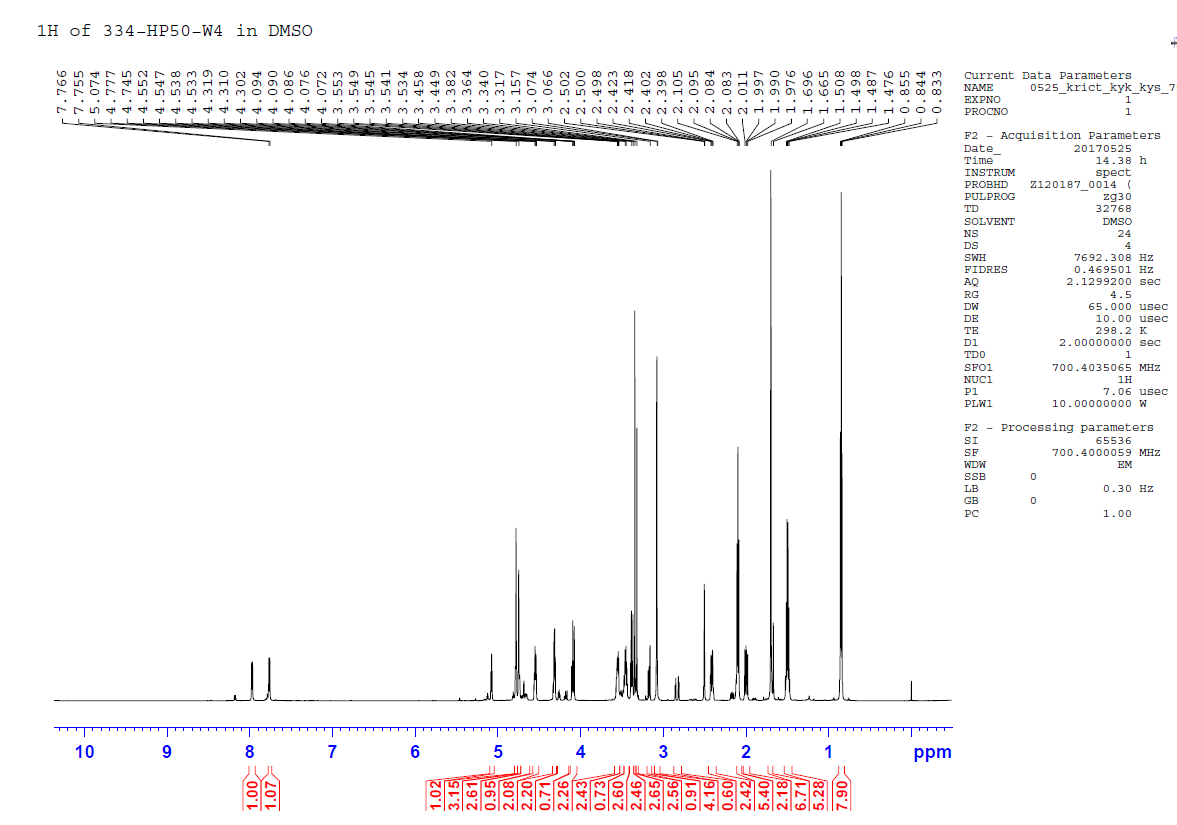


Figure S4. ^1^H-NMR (DMSO-*d*_6,_ 700 MHz) spectrum of 334-W4.


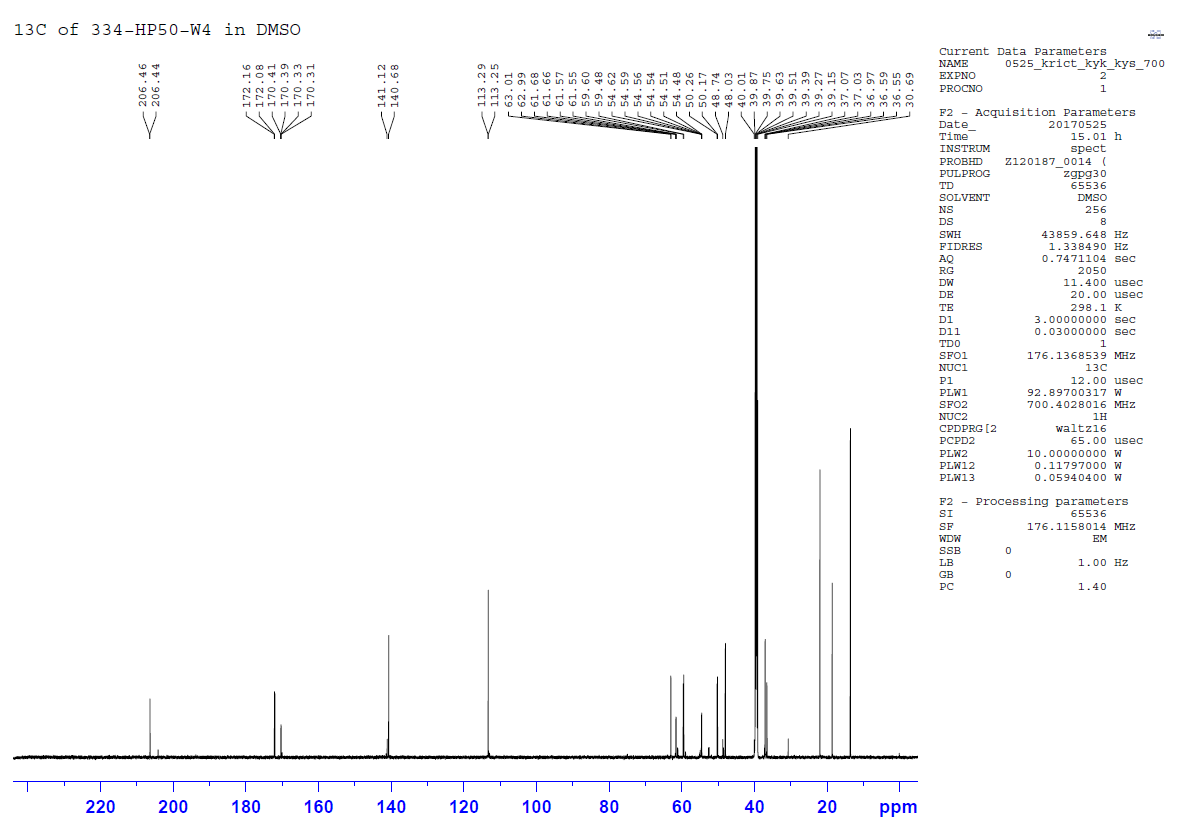


Figure S5. ^13^C-NMR (DMSO-*d*_6,_ 176 MHz) spectrum of 334-W4.


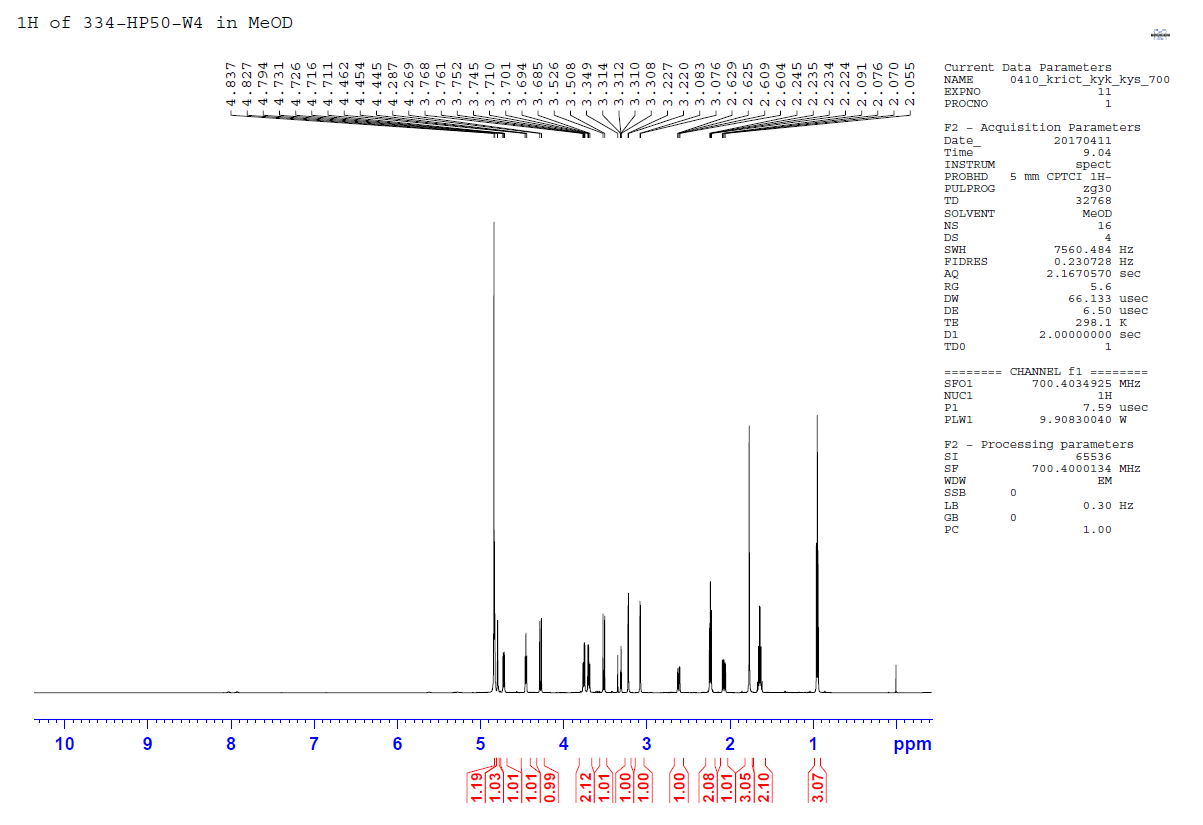


Figure S6. ^1^H-NMR (CD_3_OD_,_ 700 MHz) spectrum of 334-W4.


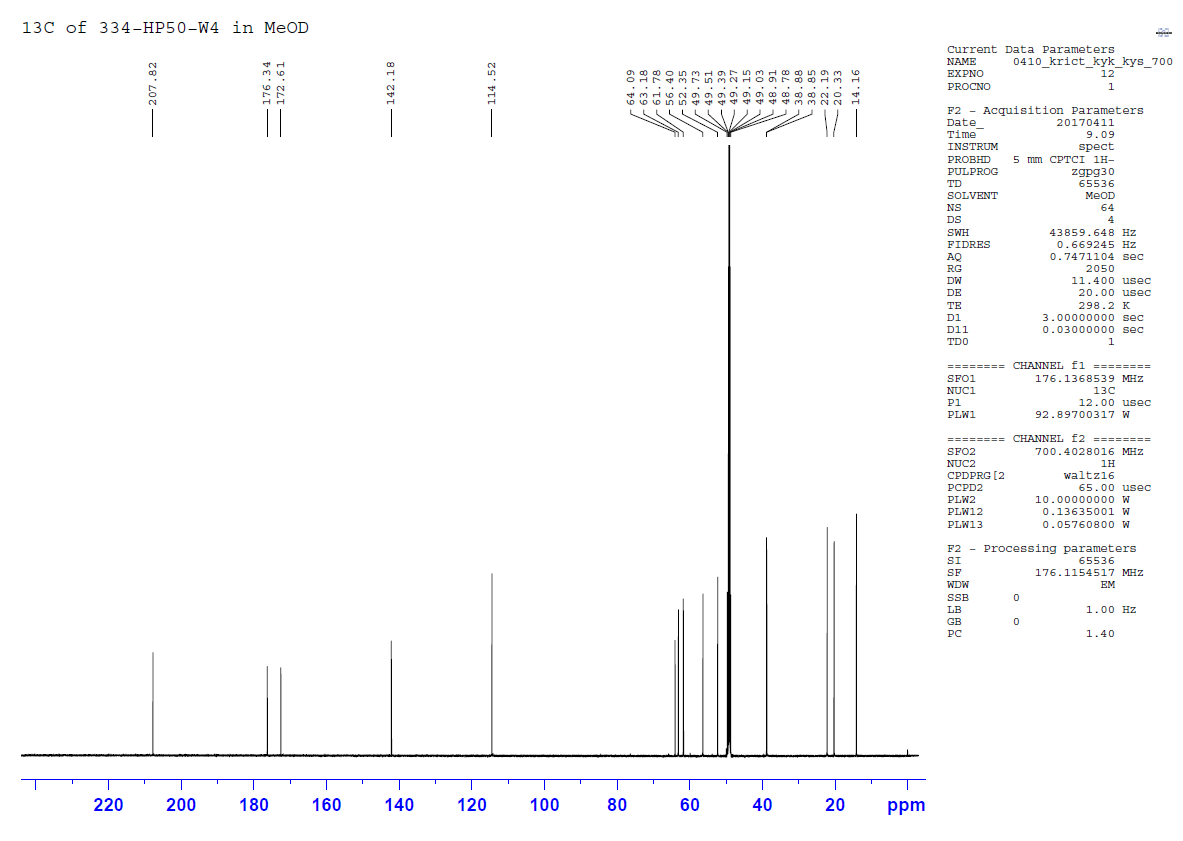


Figure S7. ^13^C-NMR (CD_3_OD_,_ 176 MHz) spectrum of 334-W4.

Figure S8. (+)ESI-MS spectrum of 334-W4.

Table S1. UV mutagenesis for active compound 334-W4 yield-up

| UV irradiation time(sec) | No. colony | Survival rate (%) |
| --- | --- | --- |
| 0 | 1113.75 ± 22.14 | - |
| 30 | 51.14 ± 3.22 | 4.58 |
| 40 | 27.23 ± 4.59 | 2.44 |
| 45 | 13.59 ±0.34 | 1.22 |
| 60 | 0.88 ± 0.14 | 0.08 |

**Table S2.** Yield change of active compound 334-W4 according to culture temperature (raw data).

| Temp.  (℃) | Concentration of 334-W4 (mg/L) | | | | | | | | | | | | | | | | | | | |
| --- | --- | --- | --- | --- | --- | --- | --- | --- | --- | --- | --- | --- | --- | --- | --- | --- | --- | --- | --- | --- |
|  | 0311-1 | | | | 0313-3 | | | | 0723-8 | | | | 0723-46 | | | | wild type | | | |
| 15 | 21.1 | 19.6 | 20.9 | **20.5** | 19.2 | 18.6 | 17.9 | **18.6** | 24.6 | 26.7 | 23.2 | **24.8** | 16.8 | 18.2 | 17.1 | **17.4** | 22.8 | 25.7 | 22.6 | **23.7** |
| 20 | 150.5 | 152.3 | 154.5 | **152.4** | 138.6 | 141.8 | 135.6 | **138.7** | 198.9 | 205.7 | 204.9 | **203.2** | 196.5 | 189.5 | 192.3 | **192.8** | 90.6 | 87.9 | 89.8 | **89.4** |
| 27 | 183.7 | 184.2 | 185.6 | **184.5** | 200.8 | 196.8 | 201.5 | **199.7** | 223.8 | 216.2 | 228.3 | **222.8** | 211.8 | 208.2 | 208.4 | **209.5** | 91.8 | 89.6 | 90.8 | **90.7** |
| 30 | 145.3 | 149.2 | 143.2 | **145.9** | 155.3 | 152.6 | 158.5 | **155.5** | 136.3 | 139.5 | 132.6 | **136.1** | 131.3 | 129.5 | 130.6 | **130.5** | 53.3 | 55.6 | 52.7 | **53.9** |
| 35 | 61.3 | 64.5 | 62.2 | **62.7** | 58.3 | 61.5 | 58.2 | **59.3** | 87.3 | 81.5 | 85.2 | **84.7** | 77.3 | 71.5 | 75.2 | **74.7** | 27.9 | 30.5 | 27.7 | **28.7** |

**Table S3.** Yield change of active compound 334-W4 according to agitation (raw data).

| RPM | Concentration of 334-W4 (mg/L) | | | | | | | | | | | | | | | | | | | |
| --- | --- | --- | --- | --- | --- | --- | --- | --- | --- | --- | --- | --- | --- | --- | --- | --- | --- | --- | --- | --- |
|  | 0311-1 | | | | 0313-3 | | | | 0723-8 | | | | 0723-46 | | | | wild type | | | |
| 50 | 0.0 | 0 | 0 | **0.0** | 0.0 | 0 | 0 | **0.0** | 0.0 | 0 | 0 | **0.0** | 0.0 | 0 | 0 | **0.0** | 0.0 | 0 | 0 | **0.0** |
| 100 | 36.4 | 32.3 | 30.1 | **32.9** | 58.6 | 51.8 | 55.2 | **55.2** | 74.2 | 76.5 | 76.8 | **75.8** | 62.5 | 64.1 | 60.3 | **62.3** | 20.6 | 18.9 | 19.8 | **19.8** |
| 150 | 182.7 | 185.2 | 185.6 | **184.5** | 189.1 | 190.3 | 186.3 | **188.6** | 230.8 | 226.8 | 233.2 | **230.3** | 214.1 | 216.5 | 219.5 | **216.7** | 66.2 | 67.9 | 69.1 | **67.7** |
| 200 | 186.3 | 189.2 | 183.6 | **186.4** | 195.4 | 195.2 | 201.1 | **197.2** | 246.3 | 243.1 | 249.5 | **246.3** | 230.3 | 229.4 | 228.5 | **229.4** | 85.7 | 89.6 | 90.6 | **88.6** |
| 250 | 161.3 | 159.2 | 160.3 | **160.3** | 209.3 | 212.5 | 207.8 | **209.9** | 264.2 | 269.5 | 259.3 | **264.3** | 245.5 | 247.8 | 249.7 | **247.7** | 86.9 | 82.1 | 85.3 | **84.8** |

**Table S4.** Yield change of active compound 334-W4 according to initial pH (raw data).

| Initial pH | Concentration of 334-W4 (mg/L) | | | | | | | | | | | | | | | | | | | |
| --- | --- | --- | --- | --- | --- | --- | --- | --- | --- | --- | --- | --- | --- | --- | --- | --- | --- | --- | --- | --- |
|  | 0311-1 | | | | 0313-3 | | | | 0723-8 | | | | 0723-46 | | | | wild type | | | |
| 3.0 | 0.0 | 0 | 0 | **0.0** | 0.0 | 0 | 0 | **0.0** | 0.0 | 0 | 0 | **0.0** | 0.0 | 0 | 0 | **0.0** | 0.0 | 0 | 0 | **0.0** |
| 5.5 | 84.6 | 82.7 | 81.2 | **82.8** | 56.2 | 53.4 | 55.7 | **55.1** | 132.2 | 126.3 | 129.1 | **129.2** | 120.5 | 121.8 | 119.3 | **120.5** | 39.0 | 37.1 | 38 | **38.0** |
| 7.0 | 192.3 | 194.3 | 197.1 | **194.6** | 198.1 | 200.3 | 197.2 | **198.5** | 192.3 | 196.4 | 191.6 | **193.4** | 176.8 | 180.2 | 180.7 | **179.2** | 95.1 | 95.4 | 98.6 | **96.4** |
| 8.5 | 203.5 | 206.7 | 205.8 | **205.3** | 204.6 | 201.3 | 201.1 | **202.3** | 233.1 | 220.8 | 224.9 | **226.3** | 198.6 | 198.4 | 195.2 | **197.4** | 99.3 | 97.2 | 101.2 | **99.2** |
| 10 | 121.0 | 125.3 | 120.1 | **122.1** | 159.4 | 160.7 | 159.6 | **159.9** | 146.2 | 142.5 | 144.1 | **144.3** | 170.6 | 171.3 | 169.8 | **170.6** | 66.9 | 64.3 | 65.8 | **65.7** |
